# Exploration of *In Vivo* and *In Vitro* Biological Effects of *Sonneratia caseolaris* (L.) Fruits Supported by Molecular Docking and ADMET Study

**DOI:** 10.1155/2023/4522446

**Published:** 2023-04-15

**Authors:** Pritam Kundu, Shovan Lal Debnath, Md. Faisal Ahad, Hiron Saraj Devnath, Lopa Saha, Utpal Kumar Karmakar, Samir Kumar Sadhu

**Affiliations:** Pharmacy Discipline, Life Science School, Khulna University, Khulna 9208, Bangladesh

## Abstract

*Sonneratia caseolaris* (L.) is a common mangrove plant which has significant medicinal value in traditional medicine. Ethanol extract from the fruits of *S. caseolaris* (SCE) was used in this project to explore its different pharmacological effects considering its traditional usage. In the castor oil-induced diarrheal method, SCE significantly lengthened the latency of the first defecation period up to 95.8 and 119.4 min as well as lowering stool count by 43.3% and 64.4% at the doses of 250 and 500 mg/kg, respectively. In evaluating the neuropharmacological effect using the open-field model, a significant central nervous system (CNS) depressant nature was observed after a reduction in the no. of squares crossed by mice at various time intervals. In evaluating the blood coagulation effect, SCE significantly reduced blood clotting time at 5.86, 5.52, and 5.01 min at 25, 50, and 100 mg/ml doses, respectively. In the assessment of the anthelmintic effect, SCE significantly killed *Paramphistomum cervi* (*P. cervi*) where the death times of the nematodes were 40.3, 36.8, and 29.9 min at 12.5, 25, and 50 mg/ml doses, respectively. The extract showed a very poor cytotoxic effect in brine shrimp lethality bioassay. In molecular docking analysis, maslinic acid, oleanolic acid, luteolin, luteolin 7-*O*-*β*-glucoside, myricetin, ellagic acid, and R-nyasol showed the best binding affinities with the selected proteins which might be the credible reasons for eliciting pharmacological responses. Among these seven compounds, only luteolin 7-*O*-*β*-glucoside had two violations in Lipinski's rule of five.

## 1. Introduction

Nature is the ultimate reservoir of all living creatures. We are always blessed by nature with its numerous medicinal plants' resources to get recuperation from many diseases since early human civilization. Although there are many updated modern techniques like combinatorial chemistry or computational drug designing system, more than half of the medicines currently used are provided by medicinal plants [1]. The use of medicinal plants in treating different ailments is as long as human civilization. Different bioactive compounds in medicinal plants provide numerous benefits to get cures for diseases. Regular consumption of different types of plant parts like fruits and vegetables protects us from many severe fatal diseases like stroke, cancer, diabetes, atherosclerosis, ulcer, and renal and neurological diseases. Moreover, due to the incessant increase in treatment, people especially having lower income are prone to take herbal medicines. So, the demand for newer and better herbal products is increasing day by day. Thus, herbal mixtures for formulating new multidimensional target drugs are now of supreme importance nowadays [2, 3].

*S. caseolaris* (L.) (cork tree) belonging to the family Sonneratiaceae is an important medicinal plant known as Choila/Ora in Bangla and crab apple in English. This plant is found in mangrove forests throughout Asia, northern Australia, and the Pacific Islands. It is known as Choila and Ora in Bangladesh and is widely available in the mangrove regions of Khulna, Satkhira, and Bagerhat districts [4, 5]. This is a long tree (height 10-15 meters) having many branches. It has cone-shaped breathing roots (pneumatophores). Its red-petalled flowers are edible and are taken as a vegetable with nampriks. The 5-7.5 cm wide round, green fruits are sour [6]. This plant is a great reservoir of numerous medicinal compounds. From its leaves, two flavonoids named luteolin and luteolin 7-*O*-*β*-glucoside were isolated by Sadhu et al. [7], while Wetwitayaklung et al. [6] reported the presence of triterpenoids and sterols. Wu et al. [5] isolated R-nyasol, R-4′-*O*-methyl nyasol, 3-hydroxy-6H-benzopyran-6-one, 3,8-dihydroxy-6H-benzopyran-6-one, benzyl-*O*-*β*-glucoside, maslinic acid, oleanolic acid, luteolin, and luteolin 7-*O*-*β*-glucoside from its fruits and by conducting high-performance liquid chromatography (HPLC) analysis. Dev et al. [8] confirmed the appearance of some polyphenolic compounds like vanillic acid, ellagic acid, and myricetin from its fruits. Most of the parts of this plant bear pharmacological significance. *S. caseolaris* ripened fruits have a pleasant scent and taste and are enriched with many vitamins. Bangladeshi people make chutney using fruits that reduce swelling whereas Thai people use those to treat cough, helminthic disease, and as blood coagulant and emollient [6, 9]. Leaves have antidiarrheal properties, and barks possess antimicrobial and antioxidant properties [9, 10]. Most of the reported scientific articles on this plant were found to be conducted on bark and leaves. Regarding the traditional uses of fruits and the existence of important medicinal constituents, we set up our target to evaluate the phytochemical and pharmacological properties. One of our previous studies reported the phytochemicals present and antioxidative, analgesic, anti-inflammatory, and antipyretic effects [11]. Upon continuation of our project, here we have reported *in vivo* antidiarrheal, CNS depressant, anthelmintic, cytotoxic, and *in vitro* blood coagulant activities along with molecular docking and ADMET study.

## 2. Materials and Methods

### 2.1. Collection, Preparation, and Authentication of Plant Materials

Matured *S. caseolaris* fruits were collected from Rampal, Bagerhat, Bangladesh, in February 2017, and dried fruit samples were identified by the experts from Bangladesh National Herbarium, Mirpur, Dhaka (accession no. DACB-43821 for future reference). In our previous paper, we reported the preparation of the extract [11]. The remaining part of that extract was used to conduct the biological tests mentioned in this manuscript.

### 2.2. Chemical and Drugs

Reagents and chemicals of analytical grade were used. Loperamide, diazepam, phytomenadione, and albendazole were procured from Square Pharmaceuticals Ltd., Bangladesh, and vincristine sulfate was purchased from Cipla Pharmaceuticals, India. Castor oil was bought from Well's Healthcare, Spain.

### 2.3. Animals

Male Swiss albino mice (*Mus musculus*) were bought from Jahangirnagar University, Bangladesh. Those were placed in the animal house for adaptation and used to conduct biological experiments mentioned in our previous literature [11]. For conducting the antidiarrheal and neuropharmacological tests reported in this manuscript, the animals were handled in the same way.

### 2.4. Evaluation of Acute Toxicity Test

To determine the toxic nature of SCE on living bodies, an acute test was conducted by the method described by Kundu et al. [12]. Doses of SCE at 1000, 3000, and 5000 milligram/kilogram (mg/kg) body weight (bw) were prepared by dissolving the sample in 1% Tween 80 in distilled water and orally administered to the different mouse groups. For the next 24 h, mice were observed and abnormalities or death of any mouse was noted for the next 7 days. In this period, specific focus was placed on observing disorders like convulsion, tremor, lethargy, and drowsiness.

### 2.5. Evaluation of Antidiarrheal Activity

Castor oil-induced diarrheal method described by Kundu et al. [12] was used to evaluate the antidiarrheal effect of SCE. Prepared solutions of SCE at 250 and 500 mg/kg and loperamide at 3 mg/kg bw doses were orally administered to specific mouse groups while the mice of the control groups were fed with 1% Tween 80 solution. After 30 minutes, each mouse was orally treated with 0.5-milliliter (ml) castor oil to induce diarrhea. Placing blotting paper in each mouse in separate cage, the no. of stools was counted up to the 4^th^ hour. The first defecation period was counted as the latent period of defecation.

The formula used to calculate the percent inhibition of diarrhea in contrast to the control group as a measure of diarrhea was given as follows:

(1) $$\begin{array}{c} \text{Inhibition of diarrhea }\left(\%\right)=\frac{\left[\left(W_{c}-W_{t}\right)\times 100\right]}{W_{c}}, \end{array}$$

where *W*_*c*_ is the average number of stools in the control group up to the 4^th^ hour and *W*_*t*_ is the average number of stools in the test group up to the 4^th^ hour.

### 2.6. Evaluation of Neuropharmacological Behavior by Open-Field Model Test

Following the open-field model described by Gupta et al. [13], the neuropharmacological nature of SCE was evaluated in a sound-attenuated room. SCE at 250 and 500 mg/kg and diazepam at 1 mg/kg bw doses were orally administered to specific mouse groups. At the same time, mice of the control groups were fed with 1% Tween 80 solution. Each animal was placed individually in any one square corner of the open-field model, and the number of squares it visited during a 3-min period on 0, 30, 60, 90, and 120 min of the research period was recorded.

### 2.7. Evaluation of Blood Coagulant Activity

The method described by Ikese et al. [14] was used to measure the *in vitro* blood coagulation activity. Serial dilution was used to create various quantities of phytomenadione (standard) and SCE. Venipuncture was used to obtain fresh human blood, and 1 ml of the blood was combined with 100 microliters (*μ*l) of prepared, varying quantities of SCE and phytomenadione. Then, these were put in a water bath at 37°C (normal human body temperature) and monitored for clot formation.

### 2.8. Evaluation of the Anthelmintic Effect

*In vivo* anthelmintic effect of SCE was assessed by a method described by Paul et al. [15]. From the freshly slaughtered oxen in local abattoirs in Khulna, live *P. cervi* were collected. 10 ml solution of SCE at 12.5, 25, and 50 mg/ml and albendazole (standard) at 15 mg/ml were prepared and put in separate Petri dishes. Six *P. cervi* were placed in each Petri dish. The paralysis time was counted when the nos. of movement of the helminthes were observed. And when the parasites did not move after vigorous shaking or being dipped in hot water (50°C), death time was recorded.

### 2.9. Evaluation of Cytotoxic Activity by Brine Shrimp Lethality Bioassay

To test the cytotoxic nature of SCE, a brine shrimp lethality bioassay was performed on nauplii of *Artemia salina* (*A. salina*) by the method described by Kundu et al. [12]. Eggs of *A. salina* were hatched to obtain nauplii. By using the serial dilution process, solutions with various concentrations of SCE and vincristine sulfate (0.1562-640 *μ*g/ml) were prepared. In every test tube, 10 alive nauplii of *A. salina* and various concentrations of SCE and vincristine sulfate samples were added and kept for the next 24 hr. After that time, the no. of alive nauplii was counted, and by using the following formula, % mortality was calculated to determine the LC_50_ (50% lethal concentration dose) value.

(2) $$\begin{array}{c} \%\text{Mortality}=\left[\frac{\left(L_{c}-L_{s}\right)}{L_{c}}\right]\times 100, \end{array}$$

where *L*_*c*_ is the average no. of alive shrimp of control solution and *L*_*s*_ is the average no. of alive shrimp of sample solution.

### 2.10. Statistical Analysis

All the experimental values presented here are expressed as average ± standard deviation (SD). A one-way analysis of variance (ANOVA) test was chosen to conduct a statistical comparison of values in the groups employed, followed by the Tukey post hoc test using SPSS (version 25). The statistical significance criterion for these findings was set at *p* < 0.05. The graphs were created using the software GraphPad Prism (version 6) [16].

#### 2.10.1. In Silico Study

*(1) Protein Preparations*. The protein models used to conduct the molecular docking studies were PBD ID: 6DDF (mu-opioid receptor), PBD ID: 6HUP (protein of gamma unit of GABA_A_), 1A3B (coagulation factor II/prothrombin), and 7ODN (tubulin-mebendazole complex) chosen and downloaded from the available data bank of proteins (http://www.rcsb.org/). The downloaded protein models were customized via Discovery Studio 2020 client (Systèmes, D. 2020. BIOVIA, Discovery Studio Visualizer, release 2019; San Diego: Dassault Systèmes) [17].

*(2) Ligand Preparations*. Three-dimensional structures of the ligands such as vanillic acid (CID: 8468), maslinic acid (CID: 73659), oleanolic acid (CID: 10494), luteolin (CID: 86289361), luteolin 7-*O*-*β*-glucoside (CID: 5319116), myricetin (CID: 5281672), ellagic acid (CID: 5281855), R-nyasol (CID: 12310493), R-4′-*O*-methyl nyasol (CID:23626544), 3,8-dihydroxy-6H-benzo[c]chromen-6-one (CID: 5488186), 3-hydroxy-6H-benzo[c]chromen-6-one (CID: 16173), benzyl glucoside (CID: 11076492), loperamide (CID: 3955), diazepam (CID: 3016), phytomenadione (CID: 5284607), and albendazole (CID: 2082) were downloaded from PubChem (https://pubchem.ncbi.nlm.nih.gov/). Finally, ligand preparation was completed, and energy minimization was carried out by PyRx [18, 19]. We decided to conduct blind docking, so no specific binding sites of the proteins were set. The grid dimensions for 6DDF, 6HUP, 1A3B, and 7ODN were *X* : *Y* : Z = 45.1318 : 53.5216 : 71.8186, 45.7974 : 43.4849 : 120.8225, 42.7474 : 46.7336 : 54.6281, and 60.2992 : 54.6657 :  69.3171, respectively.

*(3) Molecular Docking and Visualization*. The PyRx and AutoDock Vina 4.2 program was used to perform site-specific molecular docking research [18].

#### 2.10.2. Ligand-Based ADMET Prediction

Pharmacokinetic parameters of all identified compounds were carried out by considering famous Lipinski's rule of five [19]. According to this rule, a compound could show optimal drug-like behavior if it fulfills at least four of the five characteristics, namely, molecular weight < 500 Daltons, ≤ 5 hydrogen bond donors, ≤ 10 hydrogen bond acceptors, lipophilicity < 5, and molar refractivity between 40 and 130. Along with the five parameters, some other important pharmacokinetic parameters were also analyzed by using the web tools SwissADME and pkCSM, two convenient tools in drug discovery [20, 21]. Compounds that meet Lipinski's condition are considered ideal drug candidates.

## 3. Results

### 3.1. Acute Toxicity Test

No death of any mouse was observed in the observation period of 7 days on the acute toxicity test. We did not find any toxic effect on Swiss albino mice, and thus, LD_50_ could not be determined. So, we decided to conduct biological tests on mice at 250 and 500 mg/kg doses.

### 3.2. Antidiarrheal Effect

SCE significantly increased the first defecation period latency up to 95.8 and 119.4 min at 250 and 500 mg/kg doses, respectively, compared to the control group. SCE significantly reduced defecation by 43.33% and 64.44% at the applied doses whereas the standard drug loperamide showed 76.67% inhibition (Table 1).

### 3.3. CNS Depressant Effect

**Figure 1:**
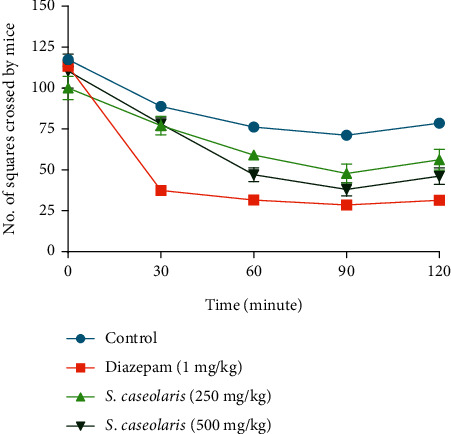
Comparison of no. of squares crossed by mice at different intervals for control, standard, and *S. caseolaris* fruit extract in the open-field model.

By the effect of SCE, mice crossed less no. of squares in the conducting period. It showed CNS depressant activity at both doses of 250 and 500 mg/kg in mice (Figure 1).

### 3.4. Blood Coagulation Effect

SCE revealed a reduction in blood clotting time. At the doses of 100, 50, and 25 mg/ml, the average clotting times were 5.016, 5.517, and 5.866 min, respectively, whereas the standard drug phytomenadione (vitamin K_1_) showed a clotting time of 3.333 min at 5 mg/ml dose. All of these values were compared to the negative control group where the clotting time was 6.72 min (Table 2).

### 3.5. Anthelmintic Effect on *P. cervi*

SCE caused both paralysis and death of the *P. cervi*. The paralysis time and death time were recorded, and those responses were obtained in a dose-dependent manner. The paralysis time was found at 39.6, 29.5, and 20.6 min at the doses of 12.5, 25, and 50 mg/ml of SCE, respectively, where 8.6 min for albendazole at the 15 mg/ml dose. On the other hand, the recorded death times for the extract were 40.3, 36.8, and 29.9 min for the respective doses and 18.5 min for the standard (Table 3).

### 3.6. Brine Shrimp Lethality Effect

**Figure 2:**
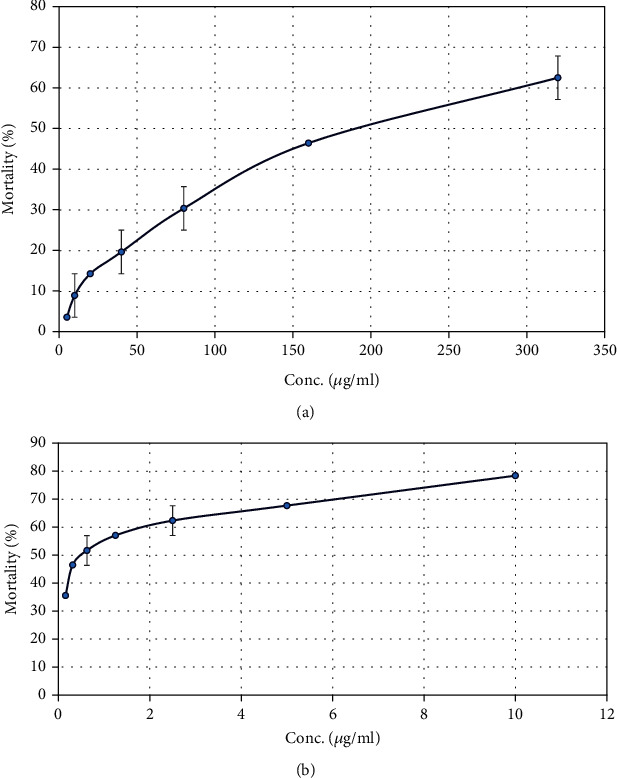
Graphical presentation of brine shrimp lethality bioassay and LC_50_ determination of (a) *S. caseolaris* fruit extract and (b) vincristine sulfate.

With an increase in concentration, SCE caused a % rise in *A. salina* nauplii mortality. The obtained LC_50_ for SCE and vincristine sulfate with these values was 219.3 and 0.584 g/ml, respectively (Figure 2).

### 3.7. Molecular Docking Study

**Figure 3:**
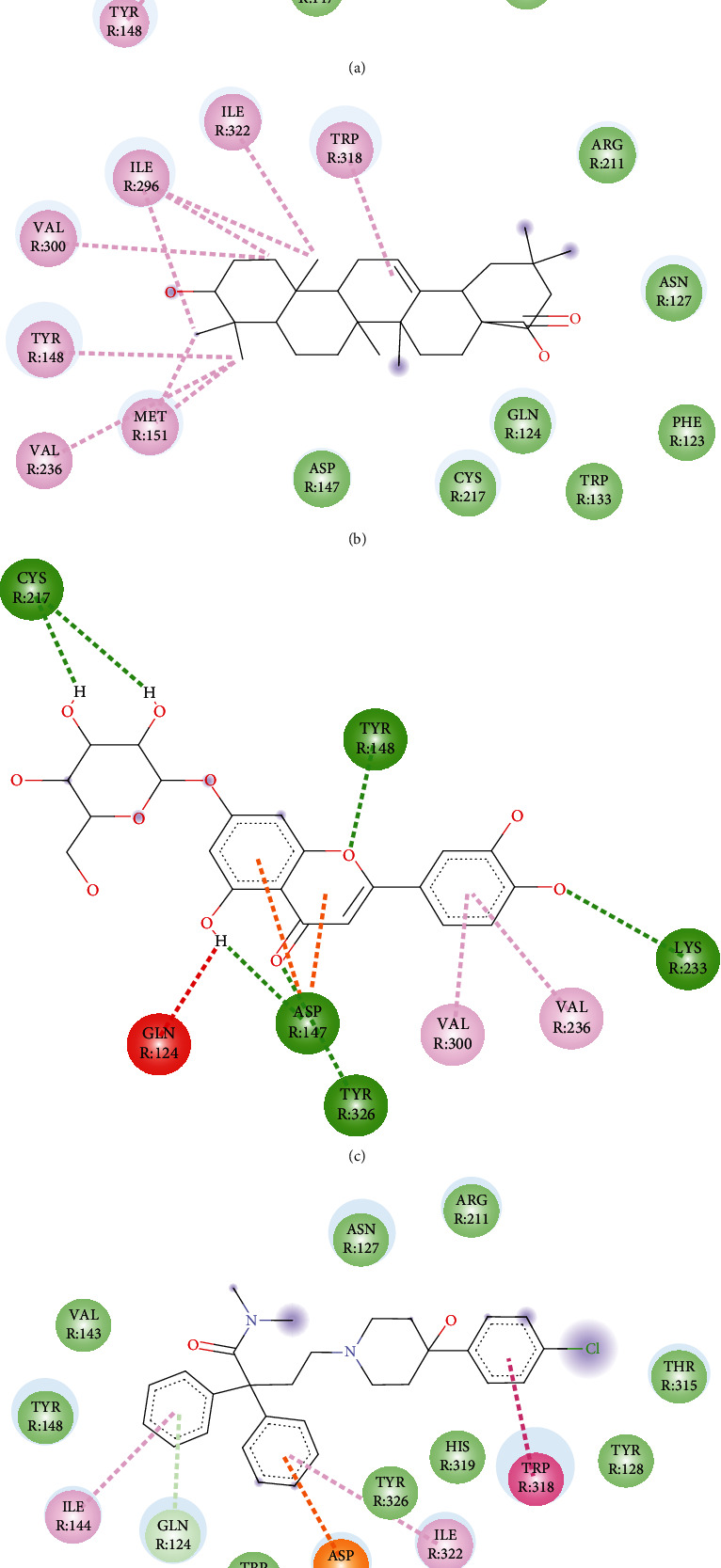
2D interactions of amino acids of 6DDF proteins with (a) maslinic acid, (b) oleanolic acid, (c) luteolin 7-O-*β*-glucoside, and (d) loperamide.

**Figure 4:**
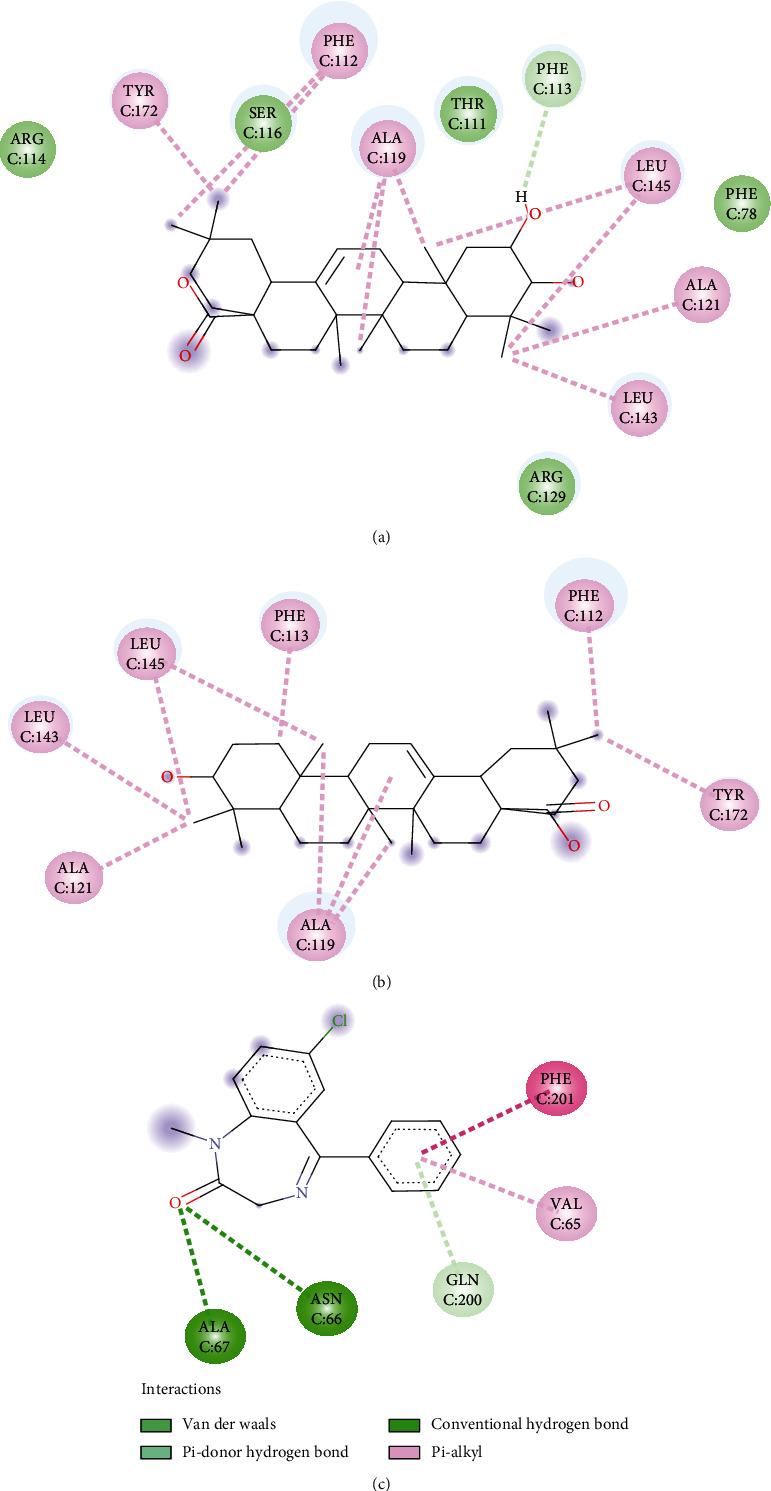
2D interactions of amino acids of 6HUP proteins with (a) maslinic acid, (b) oleanolic acid, and (c) diazepam.

**Figure 5:**
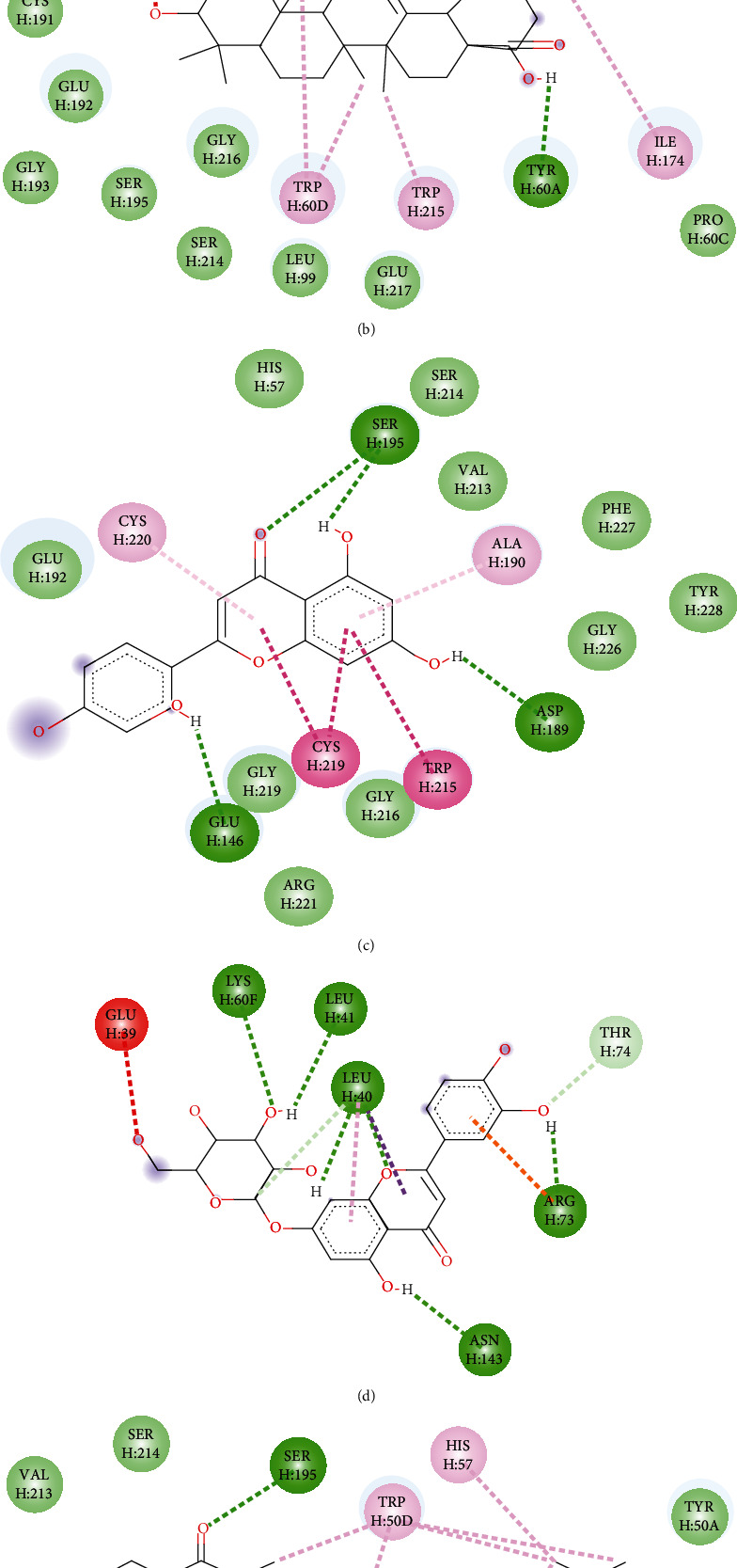
2D interactions of amino acids of 1A3B proteins with (a) maslinic acid, (b) oleanolic acid, (c) luteolin, (d) luteolin 7-*O*-*β-*glucoside, and (e) phytomenadione.

**Figure 6:**
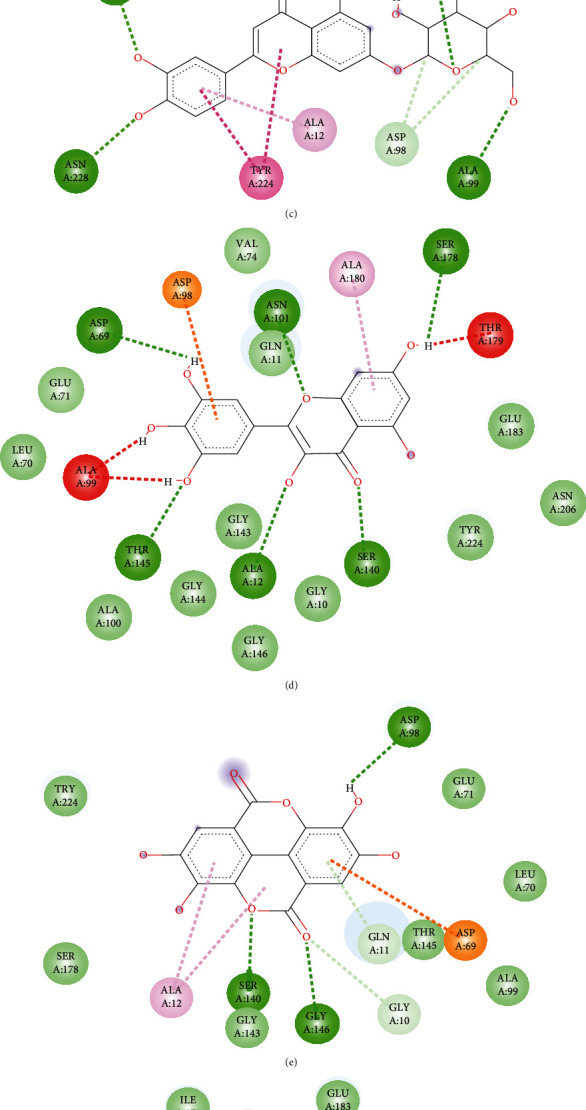
2D interactions of amino acids of 7ODN proteins with (a) oleanolic acid, (b) luteolin, (c) luteolin 7-*O-β*-glucoside, (d) myricetin, (e) ellagic acid, (f) R-nyasol, and (g) albendazole.

**Figure 7:**
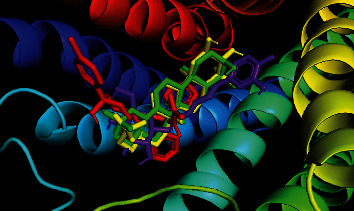
Binding pocket of maslinic acid (yellow), oleanolic acid (green), luteolin 7-*O*-*β*-glucoside (purple), and loperamide (red) with 6DDF protein.

**Figure 8:**
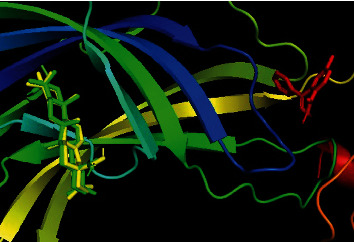
Binding pocket of maslinic acid (yellow), oleanolic acid (green), and diazepam (red) with 6HUP protein.

**Figure 9:**
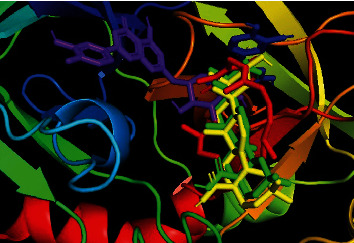
Binding pocket of maslinic acid (yellow), oleanolic acid (green), luteolin (blue), luteolin 7-*O*-*β* glucoside (purple), and phytomenadione (red) with 1A3B protein.

**Figure 10:**
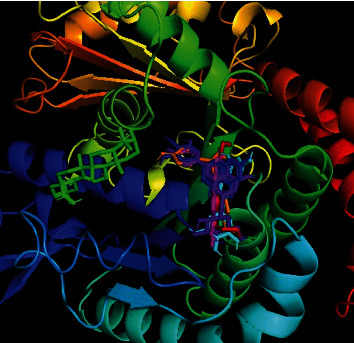
Binding pocket of oleanolic acid (green), luteolin (blue), luteolin 7-*O*-*β* glucoside (purple), myricetin (cyan), ellagic acid (magenta), R-nyasol (orange), and albendazole (red) with 7ODN protein.

Oleanolic acid and maslinic acid showed the best binding affinity (−10 kcal/mol) in the antidiarrheal test whereas that for loperamide was −8.3 kcal/mol. In the neuropharmacological test, both oleanolic acid and diazepam showed similar results (−8.3 kcal/mol and −8.2 kcal/mol). In the blood coagulant and anthelmintic tests, luteolin-7-*O*-*β*-glucoside showed the best binding affinity (−8.4 kcal/mol), and for phytomenadione and albendazole, it was −7.2 kcal/mol and −6.4 kcal/mol (Table 4). The binding of different ligands with specific amino acids of proteins is presented in Table 5. The 2D interactions of amino acids of different proteins with specific ligands are presented in Figures 3, 4, 5, and 6, and the 3D interactions among those to find out the binding regions of different ligands with amino acids of proteins are presented in Figures 7, 8, 9, and 10, respectively.

### 3.8. ADMET (Absorption, Distribution, Metabolism, Excretion, and Toxicity) and Drug-Likeliness of Ligands

All of the experimental pharmacokinetic parameters of the selected ligands are presented in Table 6. From our predicted data, we found that all of the selected ligands were lipophilic except luteolin 7-*O*-*β* glucoside while only R-nyasol was found to have blood-brain barrier (BBB) permeability. Maslinic acid and oleanolic acid were found to have a hepatotoxic nature; however, none of the compounds have any AMES toxicity. Only luteolin 7-*O*-*β* glucoside violated Lipinski's rule of five in two parameters.

## 4. Discussion

Our universe is the only entity of all living creatures. Those creatures are dependent on one another for existence, life, and many other ecological reasons. Nature has always provided the animal kingdom with a wide variety of therapeutic plants that can guard against many illnesses and pains. Numerous phytochemicals, both primary and secondary metabolites, are found in abundance in the majority of plants. The therapeutically significant substances are mostly secondary metabolites as they demonstrate a vast range of pharmacological activities that are advantageous to both plants and people. Man has always sought out newer medications from plants, from the earliest beginning to the present [22]. Each plant possesses numerous biological compounds. From the phytochemical investigation performed by Jariyah et al. [23], it was disclosed that fruits of *S. caseolaris* contain terpenoids, saponins, reducing sugars, tannins, and flavonoid polyphenolic compounds. We already reported the antioxidant activity of *S. caseolaris* fruit extract along with its anti-inflammatory effects and related in silico study [12]. From our findings, we found that SCE is highly enriched with many antioxidant compounds. A lot of polyphenolic and flavonoid compounds present in this extract such as luteolin, luteolin 7-*O*-*β*-glucoside, myricetin, and ellagic acid and triterpenoids like oleanolic acid and maslinic acid are too many important bioactive compounds which have been reported for treating numerous types of diseases like diabetes, malignancy, stroke, myalgia, anxiety, and atherosclerosis [24, 25].

Diarrhea, a common gastrointestinal disorder of tropical areas may be defined as the excretion of 3 or more slack or liquid stools in a day, is associated with increased frequency of bowel sound and movement, wet stool with or without blood, abdominal pain, and discomfort with weakness [26]. It is primarily linked to a reduction in the absorption of liquids and electrolytes or a sharp increase in intestinal motility. Castor oil's active ingredients, ricinoleic acid and nitric oxide, inhibit the Na^+^-K^+^ adenosine triphosphatase, which reduces fluid absorption and activates adenylate cyclase or mucosal cyclic adenosine monophosphate-mediated active secretion while increasing prostaglandin biosynthesis, which causes vasodilation contraction of smooth muscles and ultimately mucus secretion in the intestine [27]. In the castor oil-induced diarrheal test, SCE both increased the latency of the first defecation period and reduced the stool count. The comparative dryness, elongated frequency, and reduction in the number of stools with the extract proved its potent antidiarrheal activity. The flavonoids, luteolin, luteolin 7-*O*-*β*-glucoside [28], oleanolic acid [29], ellagic acid [30], etc., found in the plant sample may be responsible for this antidiarrheal activity. The leaves of this plant are already reported for their antidiarrheal activity, and our current finding also supports its fruits for the treatment of diarrheal disorders.

CNS, which is the most complex part of humans and other vertebrates, is the main regulatory organ of all functions in the body. CNS sometimes can be hyperexcited, and as a result, different neurological diseases arise. The development of different types of cognitive and neurological disorders is now a great threat to public health worldwide. Busy lifestyles, monotonous work, and depression lead to excess anxiety and thus aggregately affect the nervous system and lead to the generation of neurological disorders [31]. Gamma-amino-butyric acid (GABA) is the principal inhibitory neurotransmitter in the CNS. Phytoconstituents of plants, mainly different flavonoids and neuroactive steroids, are found to be active ligands for the GABA_A_ receptors in the CNS which lead to the assumption that they can behave as benzodiazepine-like molecules [32]. The open-field test is a type of experimental test that is used in scientific research to measure the general levels of locomotion, anxiety, and readiness to explore in animals (often rats). A rise in locomotor activity is a sign of excitation, whereas a decline in locomotor activity is a sign of sedation [33]. In the event of a CNS depression, the number of squares traversed by each mouse at the regular interval time will be lower, while the number of squares will be higher in the case of a CNS stimulant [13]. SCE decreased the no. of squares by mice at both 250 and 500 mg/kg doses which indicated the CNS depressant activity. The possible reason showing this effect was due to the presence of luteolin [34] and other phytochemicals like carbohydrates, glycosides, alkaloids, tannins, saponins, and flavonoids [35]. This neuroprotective activity of this plant may help treat different diseases caused by the overexcitation of neurotransmitters like seizures, panic, trauma, convulsion, and epilepsy.

Blood, the most valuable biological fluid of human and other vertebrates, carries oxygen and other essential nutrients to the cells and also returns toxic substances and carbon dioxide to be excreted from the body. Blood coagulation (clotting) is a normal defense mechanism by which blood transforms from a liquid to a gel. Blood platelets are activated, adhere to one another, and aggregate as a result of fibrin maturation, which is part of the coagulation process. After platelet aggregation and vascular spasm, prothrombinase is formed and converted into thrombin, which aids in the transformation of fibrinogen into fibrin during blood coagulation (clot) [36]. Abnormalities in blood coagulation result in thrombosis, atherosclerosis, and acute inflammation which affect a patient seriously and may be fatal. So, decreasing the prothrombin time indicates a better blood coagulant property. From our results, we can see that SCE reduced the clotting or coagulation time of blood at different doses. Compounds present in fruits like vanillic acid [37] and other phytochemicals like saponins, flavonoids, terpenoids, and steroids might be responsible for this blood coagulant property [38]. It also justifies the use of fermented fruit juice from this plant to arrest hemorrhagia [6].

Helminth infestation is one of the major prevalent infestations by parasites, which cause morbidity and mortality in animals and people worldwide. It happens mostly in undeveloped and underdeveloped countries. It is among the prominent cause of the prevalence of malnutrition, anemia, eosinophilia, and pneumonia. An unhygienic environment, lack of knowledge of sanitation, and damp weather make people susceptible to helminthiasis [39]. These parasitic worms live in the stomach of the host and secrete toxins and steal vital micronutrients from host bodies. As a result, the host losses weight, decreases milk production, and suffers from malnutrition. Medicinal plant compounds have been of great interest in the last few years for anthelmintic purposes as they contain a wide range of those types of compounds like polyphenols, tannins, and steroids [40]. SCE showed both paralyzed and lethal effects on *P. cervi*. Compounds like myricetin [41] and maslinic acid [42] in the fruits might be responsible for this anthelmintic activity, and our results also supported the plant's anthelmintic uses by Thai people as traditional medicine [6].

Cancer has become a major reason for the mortality of people worldwide, especially in the last few decades. The number and frequency of cancer-affected patients have increased to an alarming stage. Researchers have conducted a lot of research to discover potential natural anticancer agents to prevent death from this fatal disease [43]. The brine shrimp lethality assay is linked with the capability to kill laboratory-cultured *A. salina* nauplii. There lies a correlation between brine shrimp lethality bioassay and cytotoxicity [44]. In the brine shrimp lethality test, SCE did not show a notable cytotoxic effect on *A. salina* nauplii compared with the standard. The slight cytotoxic effect observed might be due to the presence of R-nyasol [45] and maslinic acid [46] in this extract, as those compounds are already reported for cytotoxic effects.

In the above *in vivo* experiments, we observed significant antidiarrheal, CNS depressant, blood coagulation, and anthelmintic effects from SCE. Henceforth, we decided to conduct in silico molecular docking study of the aforementioned activities. Molecular docking is a technique that forecasts the preferred arrangement of one molecule to another when they are linked together to form a stable complex/state. Drug formulations directly depend on the interaction between the chemicals and the receptor. In signal transduction, interactions among biologically important molecules such as nucleic acids, carbohydrates, proteins, and lipids are crucial. Molecular docking investigates the interplay between two or more molecular structures. Consequently, molecular docking helps forecast the type and amplitude of the signal generated. Characterizing binding behavior is crucial for the rational design of medications and for illuminating basic biological mechanisms [47].

For the antidiarrheal test, we selected the 6DDF protein which is a protein of *μ*-opioid receptor. The docking score of the ligand molecules in this plant along with loperamide is presented in Table 4. Our analysis found that maslinic acid and oleanolic acid showed the best binding affinities with the 6DDF protein. Among the ligands, the most common binding amino acid residues were Tyr148, Asp147, Val236, Val300, and Gln124 where Val236 was connected by Pi-alkyl bond and Tyr148, Asp147, Val300, and Gln124 were connected by the van der Waals bond (Figure 3 and Table 5). The three-dimensional binding interactions of maslinic acid, oleanolic acid, luteolin 7-O-*β*-glucoside, and loperamide with 6DDF protein are depicted in Figure 7. Our molecular docking study suggests that maslinic acid and oleanolic acid might be beneficial for exerting antidiarrheal activity, and within these, oleanolic acid [29] is already reported for this effect.

In the neuropharmacological test, the selected protein was 6HUP, a protein of gamma unit of GABA_A_. Docking scores of the ligands along with diazepam are presented in Table 4. Our analysis found that oleanolic acid and maslinic acid showed the best binding affinities with the 6HUP protein. Among the ligands, the most common binding amino acid residues were Ala119, Ala121, Leu143, Leu145, Phe112, Phe113, and Tyr172, all of which formed Pi-alkyl bond (Figure 4 and Table 5). The three-dimensional binding interactions of maslinic acid, oleanolic acid, and diazepam with 6HUP protein are represented in Figure 8. Although maslinic acid and oleanolic acid were bound in the same region, diazepam bonded to a separate one. Thus, it might be suggested that these two triterpenoids might be a good target for obtaining new CNS depressant drugs.

In the blood coagulation test, the protein 1A3B of alpha thrombin (coagulation factor II) was selected for docking analysis. Docking scores of the ligands along with diazepam are presented in Table 4. Our analysis found that oleanolic acid, maslinic acid, luteolin, and luteolin 7-*O*-*β*-glucoside showed the best binding affinities with 1A3B protein. Among the ligands, the most common binding amino acid residues were Ser195, Ser124, Gly216, Gly219, Cys191, and Cys220. Ser195 formed a conventional H bond, Cys191 and Cys220 formed Pi-alkyl bonds, and the other Ser214, Gly126, and Gly219 formed the van der Waals bonds (Figure 5 and Table 5). The three-dimensional binding interactions of oleanolic acid, maslinic acid, luteolin, and luteolin 7-*O*-*β*-glucoside and phytomenadione with 1A3B protein are portrayed in Figure 9. Only vanillic acid [37] in this plant is reported for blood coagulation effect, and in silico analysis gives us an assumption that triterpenoids like maslinic acid and oleanolic acid and flavonoids like luteolin and luteolin 7-*O-β*-glucoside might be good targets to obtain newer blood coagulants.

In the anthelmintic test, the selected protein was 7ODN, a protein of tubulin. Docking scores of the ligands along with diazepam are presented in Table 4. From our analysis, we found that oleanolic acid, luteolin, luteolin 7-*O*-*β*-glucoside, R-nyasol, and ellagic acid showed the best binding affinities with the 7ODN protein. Among the ligands, the most common binding amino acid residues are Tyr224, Ala12, Ala99, Glu183, Asn206, and Glu11. Tyr224 and Ala12 formed Pi-Pi stacked bonds, and the other four amino acids Ala99, Glu183, Asn206, and Glu11 formed the van der Waals bonds (Figure 6 and Table 5). The three-dimensional binding interactions of oleanolic acid, luteolin, luteolin 7-*O*-*β*-glucoside, R-nyasol, ellagic acid, and albendazole with 7ODN protein are shown in Figure 10. These compounds might be good targets to synthesize newer anthelmintic drugs, and among them, myricetin [41] is already reported for this activity.

**Figure 11:**
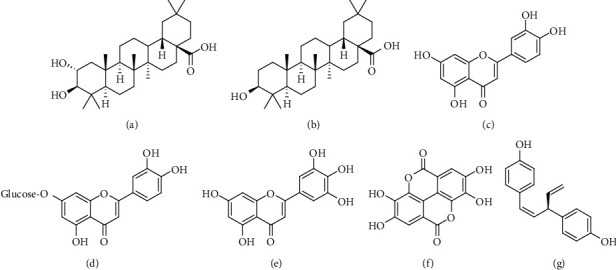
Chemical structures of (a) maslinic acid, (b) oleanolic acid, (c) luteolin, (d) luteolin 7-*O*-*β*-glucoside, (e) myricetin, (f) ellagic acid, and (g) R-nyasol.

From the molecular docking study, we can summarize that some important compounds in the SCE mainly maslinic acid, oleanolic acid, luteolin, luteolin 7-*O*-*β*-glucoside, myricetin, ellagic acid, and R-nyasol might be responsible for eliciting the related pharmacological effects (Figure 11). Further research on these striking molecules is recommended.

The ADMET study was conducted to explore the safety margin of the ligands that showed the best docking scores in the molecular docking study. Our results suggest that maslinic acid, oleanolic acid, luteolin, myricetin, ellagic acid, and R-nyasol are found to be safe to use as drug products as those compounds did not violate Lipinski's rule of five (not more than one violation). However, in the case of luteolin 7-*O*-*β*-glucoside, there were two violations of Lipinski's rule of five.

## 5. Conclusion

This research was conducted on *S. caseolaris* fruit to explore its pharmacological effects relying on the traditional usage of this plant. The crude extract elicited very promising antidiarrheal, sedative, blood coagulant, and anthelmintic effects without any cytotoxic nature. The observed responses were due to the presence of diverse phytochemical groups and bioactive metabolites which justify its folklore medicinal usage. Our pharmacological findings along with computer-aided analysis might be helpful for natural product scientists to discover suitable lead molecules for various ailments as a part of drug discovery.

## Figures and Tables

**Table 1:** Representation of effects of *S. caseolaris* fruit extract on castor oil-induced diarrhea in mice.

Treatment group	Dose (mg/kg)	Average latent period of defecation	% increase in latent period of defecation	Average no. of stool	% inhibition of defecation
Control	—	32 ± 4.527^*θ*▲Δ^	—	18 ± 2.00^*θ*▲Δ^	—
Loperamide	3	171 ± 14.195^∗^^▲Δ^	434.37^∗^^▲Δ^	4.2 ± 1.30^∗^^▲^	76.67^∗^^▲^
SCE	250	95.8 ± 7.049^∗^^*θ*Δ^	199.37^∗^^*θ*Δ^	10.2 ± 0.86^∗^^*θ*Δ^	43.33^∗^^*θ*Δ^
SCE	500	119.4 ± 14.046^∗^^*θ*▲^	273.12^∗^^*θ*▲^	6.4 ± 1.14^∗^^▲^	64.44^∗^^▲^

Data are expressed as means of five replicates ± SD (standard deviation); ^∗^*p* < 0.05 vs. control (Dunnett's *t*-test); ^*θ*^*p* < 0.05 vs. loperamide 3 mg/kg; ^▲^*p* < 0.05 vs. SCE 250 mg/kg; ^Δ^*p* < 0.05 vs. SCE 500 mg/kg (pair-wise comparison by post hoc Tukey's test).

**Table 2:** Representation of blood coagulation effects of *S. caseolaris* fruit extract.

Treatment group	Dose (mg/ml)	Mean coagulation time (min) ± SD
Control	—	6.717 ± 0.212^*θ*▲Δ●^
Standard (phytomenadione)	5	3.333 ± 0.071^∗^^▲Δ●^
SCE	25	5.866 ± 0.94^∗^^*θ*Δ●^
SCE	50	5.517 ± 0.141^∗^^*θ*▲●^
SCE	100	5.016 ± 0.047^∗^^*θ*▲Δ^

Data are expressed as means of five replicates ± SD (standard deviation); ^∗^*p* < 0.05 vs. control (Dunnett's *t*-test); ^*θ*^*p* < 0.05 vs. phytomenadione 5 mg/ml; ^▲^*p* < 0.05 vs. SCE 25 mg/ml; ^Δ^*p* < 0.05 vs. SCE 50 mg/ml; ^●^*p* < 0.05 vs. SCE 100 mg/ml (pair-wise comparison by post hoc Tukey's test).

**Table 3:** Representation of anthelmintic effects of *S. caseolaris* fruit extract.

Treatment group	Dose (mg/ml)	Mean paralysis time (min)	Mean death time (min)
Control	—	—	—
Albendazole	15	8.6 ± 0.8^▲Δ^^∗^	18.57 ± 0.89^▲Δ^^∗^
SCE	12.5	39.62 ± 1.45^*θ*Δ^^∗^	40.34 ± 0.61^*θ*Δ^^∗^
SCE	25	29.59 ± 1.03^*θ*▲^^∗^	36.83 ± 0.66^*θ*▲^^∗^
SCE	50	20.65 ± 1.2^*θ*▲Δ^	29.98 ± 1.45^*θ*▲Δ^

Data are expressed as means of six replicates ± SD (standard deviation); ^*θ*^*p* < 0.05 vs. albendazole 15 mg/ml; ^▲^*p* < 0.05 vs. SCE 12.5 mg/ml; ^Δ^*p* < 0.05 vs. SCE 25 mg/ml; ^∗^*p* < 0.05 vs. SCE 50 mg/ml (pair-wise comparison by post hoc Tukey's test).

**Table 4:** Binding affinities of all ligands against 6DDF, 6HUP, 1A3B, and 7ODN proteins.

Ligand name	PubChem ID	6DDF (mu-opioid receptor)	6HUP (gamma unit of GABA_A_)	1A3B (coagulation factor II/prothrombin)	7ODN (tubulin)
Maslinic acid	73659	−**10**	−**8.1**	−**8.2**	−7.9
Oleanolic acid	10494	−**10**	−**8.3**	−**8.2**	−**8.0**
Luteolin	5280445	−7.3	−6.8	−**8.1**	−**8.8**
Luteolin 7-*O-β*-glucoside	86289361	−**8.3**	−7.9	−**8.4**	−**10.4**
Myricetin	5281672	−7.2	−6.8	−7.8	−**9.0**
Ellagic acid	5281855	−7.5	−7.5	−7.8	−**8.8**
R-Nyasol	6438674	−7.2	−7.4	−7.7	−**8.0**
R-4′-*O*-methyl nyasol	23626544	−7.6	−6.9	−6.8	−7.3
3,8-Dihydroxy-6H-benzo[b,d]pyran-6-one	5488186	−6.7	−7.6	−7	−7.8
3-Hydroxy-6H-benzo[b,d]pyran-6-one	16173	−7.1	−7.6	−7	−7.7
Benzyl glucopyranoside	11676492	−6.8	−6.1	−7.2	−7.5
Vanillic acid	8468	−5.1	−6.5	−5.7	−6
Loperamide	3955	−**8.3**			
Diazepam	3016		**-8.2**		
Phytomenadione	5284607	—	—	−7.2	—
Albendazole	2082				−6.4

Compounds showing best binding affinities (< −8 kcal/mol) are depicted in bold style.

**Table 5:** Binding of different ligands with amino acids of different proteins.

Ligands	Proteins	Interacting amino acids
Loperamide	6DDF	Asn127, Arg244, Thr315, Tyr128, Trp318, His319, Ile144, Ile322, **Tyr148**, Tyr326, **Asp147**, Trp133, **Gln124**, Val143
Maslinic acid		Ile296, Ile322, **Val236**, **Val300**, Trp318, Arg211, Thr218, Asp216, **Asp147**, Asn127, Cys217, **Gln124**, Trp133, **Tyr148**, Met151, His297
Oleanolic acid		Ile296, Ile322, Trp133, Trp318, **Gln124**, Cys217, Arg211, Asn127, Phe123, **Asp147**, Met151, **Val236**, **Val300**, **Tyr148**
Luteolin 7-*O*-*β-*glucoside		Cys217, **Tyr148**, Lys233, **Val236**, **Val300**, Tyr326, **Asp147**, **Gln124**

Diazepam	6HUP	Ala67, Asn66, Gln200, Val65, Phe201
Oleanolic acid		**Ala119**, **Ala121**, **Leu143**, **Leu145**, **Phe112**, **Phe113**, **Tyr172**
Maslinic acid		**Ala119**, **Ala121**, **Leu143**, **Leu145**, Phe78, **Phe112**, **Phe113**, **Tyr172**, Arg114, Arg129, Ser116, Thr111

Phytomenadione	1A3B	Val213, **Ser195**, **Ser214**, Trp60D, Trp215, His57, Tyr60A, Ala190, **Cys191**, **Cys220**, **Glu192**, Glu216, **Gly216**, **Gly219**, Leu99, Ile174, Asn98
Maslinic acid		Pro60C, Glu97A, Tyr60A, **Gly216**, **Gly219**, **Ser195**, **Ser214**, **Cys191**, **Cys220**, **Glu192**, Ile174, Leu99, Trp215, Trp60D
Oleanolic acid		**Cys220**, **Cys191**, **Gly216**, **Gly219**, Gly193, **Glu192**, Glu217, **Ser195**, **Ser214**, Trp60D, Trp215, Ile174, Pro60C, Leu99, Tyr60A
Luteolin		Ala190, Asp189, Arg221, **Ser195**, **Ser214**, Glu146, **Glu192**, Glu219, **Gly216**, **Gly219**, Gly226, **Cys191**, **Cys220**, Phe227, Tyr228, Val213, His57
Luteolin 7-*O-β*-glucoside		Glu39, Lys60F, Leu41, Leu40, Thr74, Arg73, Asn143

Albendazole	7ODN	**Ala12**, **Ala99**, Glu143, **Glu183**, Ser140, **Asn206**, **Tyr224**
Oleanolic acid		Asn18, Glu77, Gly81, Gln15, Thr82, Thr223, Thr225, Tyr 83, **Tyr224**, Val78
Luteolin		**Ala12**, Ala180, Asn101, Asn228, **Asn206**, Gly143, **Gln11**, Gln15, **Glu183**, Ile16, Ile171, Ile231, Thr179, **Tyr224**, Ser178, Leu227
Luteolin 7-*O*-*β*-glucoside		**Ala12**, **Ala99**, Asn101, **Asn206**, Asn228, Asp98, Glu71, **Glu183**, **Gln11**, **Tyr224**
Myricetin		Ala12, Ala99, Ala100, Ala180, Asn101, **Asn206**, Asp69, Asp98, Gly10, Gly143, Gly144, Gly146, **Gln11**, Glu71, **Glu183**, Ile16, Thr145, Thr179, **Tyr224**, Ser178, Leu70, Val74
Ellagic acid		**Ala12**, **Ala99**, Gly10, Gly143, Gly146, Asp69, Asp98, Glu71, **Gln11**, Thr145, Leu70, **Tyr224**, Ser140, Ser178
R-Nyasol		**Ala12**, **Ala99**, Asn101, **Asn206**, Asn228, **Gln11**, Gln15, Gly10, Gly142, Gly143, Gly144, Gly146, Thr145, **Tyr224**, Ile16, Ile171, **Glu183**, Ser140

Amino acids common for ligands to a particular protein are depicted in bold style.

**Table 6:** ADMET analysis of selected ligands.

Name of the ligands	Maslinic acid	Oleanolic acid	Luteolin	Luteolin 7-*O*-*β* glucoside	Myricetin	Ellagic acid	R-Nyasol
MW (g/mol)	472.70	456.70	286.24	448.38	318.24	302.19	252.31
NRB	1	1	1	4	1	0	4
NHA	4	3	6	11	8	8	2
NHD	3	2	4	7	6	4	2
MR	137.82	136.65	76.01	108.13	80.06	75.31	79.01
TPSA (Å^2^)	77.76	57.53	111.13	190.28	151.59	141.34	40.46
logP	6.2044	7.2336	2.2824	−0.2445	1.6936	1.3128	4.0808
BBBP	No	No	No	No	No	No	Yes
IA (% absorbed)	100	99.931	81.13	16.136	65.93	86.684	92.615
TC (log ml/min/kg)	−0.071	−0.081	0.0495	0.633	0.422	0.537	0.217
LD_50_ (mol/kg)	2.516	2.349	2.455	2.599	2.497	2.399	2.27
MTD (log mg/kg/day)	0.127	0.203	0.499	0.0803	0.51	0.476	0.117
HT	Yes	Yes	No	No	No	No	No
AT	No	No	No	No	No	No	No
NLV	1	1	0	2	1	0	0
DL	Yes	Yes	Yes	No	Yes	Yes	Yes

MW: molecular weight; NRB: no. of rotatable bonds; NHA: no. of hydrogen bond acceptor; NHD: no. of hydrogen bond donor; MR: molar refractivity; TPSA: topological polar surface area; logP: predicted octanol/water partition coefficient; BBBP: blood-brain barrier permeability; IA: intestinal absorption (% absorbed); TC: total clearance; LD_50_: oral rat acute toxicity; MTD: maximum tolerated dose for human; HT: hepatotoxicity; AT: AMES toxicity; NLV: number of Lipinski's violation; DL: drug-likeness.

## Data Availability

All experimental data of different conducted tests in this manuscript are stored on our computer, and these will be provided upon request or whenever needed.
